# Development and Technical Validation of a Smartphone-Based Cry Detection Algorithm

**DOI:** 10.3389/fped.2021.651356

**Published:** 2021-04-13

**Authors:** Ahnjili ZhuParris, Matthijs D. Kruizinga, Max van Gent, Eva Dessing, Vasileios Exadaktylos, Robert Jan Doll, Frederik E. Stuurman, Gertjan A. Driessen, Adam F. Cohen

**Affiliations:** ^1^Centre for Human Drug Research, Leiden, Netherlands; ^2^Juliana Children's Hospital, Haga Teaching Hospital, Hague, Netherlands; ^3^Leiden University Medical Centre, Leiden, Netherlands; ^4^Department of Pediatrics, Maastricht University Medical Centre, Maastricht, Netherlands

**Keywords:** crying, infant, home-monitoring, hospital-monitoring, smartphone, machine learning

## Abstract

**Introduction:** The duration and frequency of crying of an infant can be indicative of its health. Manual tracking and labeling of crying is laborious, subjective, and sometimes inaccurate. The aim of this study was to develop and technically validate a smartphone-based algorithm able to automatically detect crying.

**Methods:** For the development of the algorithm a training dataset containing 897 5-s clips of crying infants and 1,263 clips of non-crying infants and common domestic sounds was assembled from various online sources. OpenSMILE software was used to extract 1,591 audio features per audio clip. A random forest classifying algorithm was fitted to identify crying from non-crying in each audio clip. For the validation of the algorithm, an independent dataset consisting of real-life recordings of 15 infants was used. A 29-min audio clip was analyzed repeatedly and under differing circumstances to determine the intra- and inter- device repeatability and robustness of the algorithm.

**Results:** The algorithm obtained an accuracy of 94% in the training dataset and 99% in the validation dataset. The sensitivity in the validation dataset was 83%, with a specificity of 99% and a positive- and negative predictive value of 75 and 100%, respectively. Reliability of the algorithm appeared to be robust within- and across devices, and the performance was robust to distance from the sound source and barriers between the sound source and the microphone.

**Conclusion:** The algorithm was accurate in detecting cry duration and was robust to various changes in ambient settings.

## Introduction

Crying is a primary indicator of decreased infant well-being (1). Besides the normal crying-behavior that is natural for every infant, a change in cry duration, intensity or pitch can be a symptom of illness (2). Cry duration has been used as a biomarker for diagnostic and follow-up purposes for a wide range of clinical conditions of infancy, such as gastroesophageal reflux and cow milk allergy (3, 4). However, traditional methods to record cry behavior, such as parent- or nurse- reported cry duration, are subjective and vulnerable to observer bias (5). On the other hand, more objective manual annotating of audio recordings is labor intensive and may be subject to privacy-concerns by parents. An objective, automated and unobtrusive method to quantify crying behavior in an at-home and clinical setting may improve the diagnostic process in excessively crying infants, allow for objective determination of treatment effects by physicians, and enable researchers to include objectively determined cry duration as digital biomarker in clinical trials. Therefore, a classification algorithm is necessary for the automatic recognition of cries in audio files. Given the importance for researchers to study the relationship between an infant's crying patterns and their health, automatic detection and quantification of infant cries from an audio signal is an essential step in remote baby monitoring applications (6).

Automatic cry detection has been reported in the form of remote baby monitors for non-intrusive clinical assessments of infants in hospital settings (6–9), and several researchers have shown that classification of cry- and non-cry-sounds is possible with machine-learning algorithms (10–12). However, most algorithms lack validation in a completely independent dataset, which is crucial to predict performance in new- and real-world settings, while data regarding intra- and inter-device variability and other factors that may influence repeatability is lacking as well (10, 13, 14). Finally, algorithms are often developed for use on personal computers or dedicated devices. Usability of an algorithm would be increased if it were available on low-cost consumer-devices such as smartphones, which are readily available in most households and are easy to operate. Furthermore, smartphones have adequate processing power to analyse and transmit data continuously for monitoring in real-time. The aim of this study was to develop and validate a smartphone-based cry-detection algorithm that is accurate, reliable, and robust to changes in ambient conditions.

## Materials and Methods

### Location and Ethics

This was a prospective study conducted by the Center for Human Drug Research (CHDR) and Juliana Children's Hospital. The study protocol was submitted to the Medical Ethics Committee Zuidwest Holland (ID 19-003, Leiden, Netherlands), who judged the protocol did not fall under the purview of the Dutch Law for Research with Human Subjects (WMO). The study was conducted in compliance with the General data protection regulation (GDPR). The algorithm was developed and reported in accordance with EQUATOR guidelines (15).

### Algorithm Development

#### Training Dataset

A training dataset was obtained from various online sources (Supplementary Table 2) and consisted of both crying- and non-crying sounds. Non-crying sounds consisted of common real-life sounds and included talking, breathing, footsteps, cats, sirens, dogs barking, cars honking, snoring, glass breaking, and ringing of church clocks. Furthermore, non-crying infant sounds (hiccoughs, wailing, yelling, babbling, gurgles, and squeaking), as well as adult crying sounds, were included in the training dataset. All sounds were played back through a loudspeaker and processed into non-overlapping 5-s epochs on a G5 (Motorola, Chicago, IL, USA) or G6 (Motorola, Chicago, IL, USA) smartphones and. A total of 1,591 audio features (Supplementary Text 3) were extracted from each 5-s epoch with openSMILE (version 2.3.0, audEERING, Gilching, Germany) (16) on the smartphone. Each 5-s epoch was manually annotated as crying or non-crying by a single investigator. A 5-s epoch was selected due to the fact that the median cry duration (without a silent break) in the training dataset was 4 s.

#### Algorithm Training

To prevent overfitting of the algorithm on non-robust audio features provide by the software, manual feature selection was performed to exclude features that exhibited different distributions when analyzed under different conditions (Supplementary Text 3). Feature selection was performed using the audio file generated during the robustness tests. The file was played back through a laptop speaker during differing ambient conditions with (see paragraph Robustness-tests in section Materials and Methods), a dedicated speaker, and processed to openSMILE features with the CHDR MORE® application. Additionally, the raw file was processed using openSMILE software on a personal computer. Considering the data was derived from the exact same audio file, the distribution of features should be identical during all conditions (Supplementary Text 3). However, this was not the case for all features, particularly those that were derived from the extremes of each feature (e.g., Percentile 1% percentile 99%). Therefore, distribution plots were judged visually by the authors and each feature that demonstrated a clear difference in means or standard deviations across conditions was excluded from the final dataset. After selection, 980 features audio features remained in the dataset. Two discriminative classifiers [Random Forest and Logistic Regression (17–20)] and one generative classifier (Naïve Bayes) were considered for the classification of crying and non-crying sounds. For each classifier, a 5-fold cross-validated grid-search to select the best combination of features and hyper-parameters was performed to minimize the error estimates in the final model. The primary objective of the model was to identify crying and therefore, hyper-parameters that optimized for sensitivity were prioritized. This was followed by 5-fold cross-validation to robustly estimate the model performance and generalization of the model. The classifier with the highest Matthew's Correlation Coefficient (MCC) was chosen as the final model and subjected to algorithm validation.

### Algorithm Validation

#### Data Collection

An independent validation dataset was obtained from two sources. First, audio recordings were made in an at-home setting of 4 babies aged 0–6 months using the G5 or G6 smartphones. Second, audio recordings were made with the G5 or G6 smartphones of 11 babies aged 0–6 months admitted to the pediatric ward due to various reasons. Audio recordings were made after obtaining informed consent from both parents and were stripped of medical- and personal information prior to analysis.

#### Performance Analysis

Each 5-s epoch in the recordings was annotated as crying- and non-crying by one annotator. In the case of doubt on how to classify an epoch, two additional annotators were included, and a choice was made *via* blinded majority voting. The developed algorithm was used to classify each epoch, and annotations and classifications were compared to calculate the accuracy, MCC, sensitivity, specificity, positive predictive value (PPV) and negative predictive value (NPV) in the complete dataset and in the hospital- and home datasets separately.

#### Post-processing of Cry Epochs Into Novel Biomarkers

Some infants are reported to cry often, but with short intervals in between. Only counting the number of epochs that contain crying for such infants could result in an underestimation of the burden for infants and parents. As such, the duration of “cry sequences” (periods during which an infant is crying either continuously or occasionally) is an important additional feature. To calculate this, post-processing of detected cries was performed to calculate the number and duration of cry sequences as separate candidate biomarkers. A cry sequence was defined by the authors with a start criterion (at least six 5-s epochs containing crying within 1 min) and a stop criterion (no crying detected for 5 min). Individual timelines were constructed for true- and predicted cry sequences to determine the reliability of the algorithm for this novel biomarker.

#### Robustness Tests

A series of robustness tests was conducted to ensure that the developed algorithm was robust to varying conditions when used with a smartphone with the final application (CHDR MORE®) installed, which is how the algorithm would be deployed in practice. A 29-min-long clip containing 16.7 min of crying was played from a speaker with a smartphone with the CHDR MORE® application in proximity. This application, developed in-house, has incorporated openSMILE technology and is able to extract and transmit audio features. The following conditions were tested during this phase of the study: intra-device variability (*n* = 10), inter-device variability (*n* = 10), distance from audio source (0.5, 1, 2, and 4 m) and by placing the phone behind several barriers and in the presence of background tv sounds. For intra-device variability, a single phone was used 10 times to determine repeatability within a single device. For inter-device variability, 10 different devices of the same type (G6) were used to determine the repeatability across devices. Because it was not technically possible to pair the application output with the raw audio features of the original recording, cumulative cry count plots were construed for each condition and compared with cumulative cries in the original recording.

A schematic overview of the analysis steps is displayed in Supplementary Figure 1.

## Results

### Algorithm Training

The training set consisted of 897 5-s audio clips, as well as 1,263 non-crying 5-s clips. Of the three methods applied to develop the algorithm, the Random Forest method achieved the highest accuracy and MCC with 93.8 and 87.3%, respectively (Table 1). The 10 most important audio features for the algorithm were derived from Mel Frequency cepstral coefficients, Mel frequency bands and Voicing Probability. A variable importance plot of the 10 most important features included in the final algorithm is displayed in Supplementary Figure 4.

**Table 1:** Performance of the final algorithm.

**Training dataset**		**Validation dataset**		
**Parameter**	Performance [Mean (SD)]^*^	**Hospital subjects (*n* = 11) (%)**	**Home subjects (*n* = 4) (%)**	**All subjects (*n*= 15) (%)**
Accuracy	93.8% (±1.1%)	98.5	99.7	98.7
MCC	87.3% (±2.2%)	75.5	98.6	78.4
Sensitivity	93.8% (±1.1%)	80.6	97.5	83.2
Specificity	94.8% (±1.1%)	99.1	100	99.2
PPV	-	72.2	100	75.2
NPV	-	99.4	99.6	99.5

*MCC, Matthew's Correlation Coefficient; PPV, positive predictive value; NPV, negative predictive value*.

* *Mean (SD) performance of 5-fold cross validation*.

### Algorithm Validation

**Figure 1:**
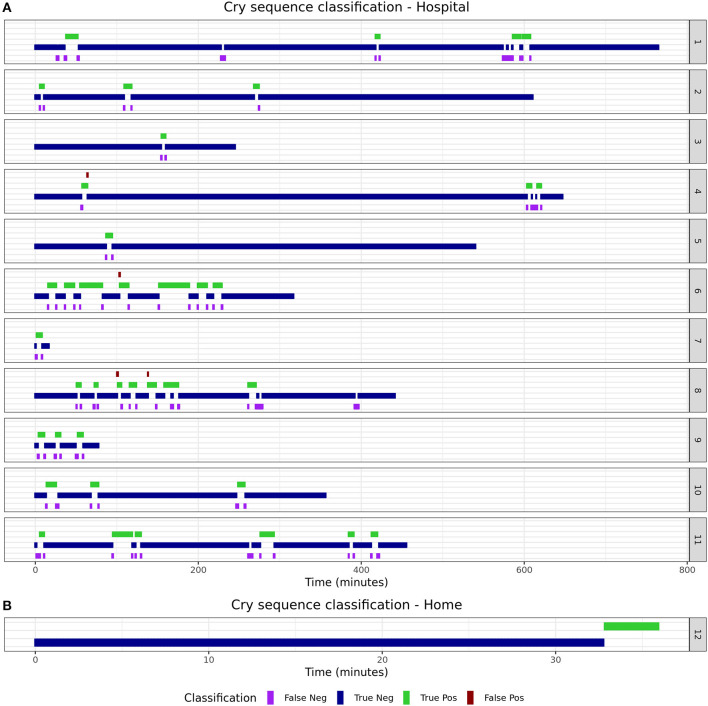
True and predicted cry sequence per infant.

The 15 infants [mean age: 2 months (SD 1.9)] created a total of 150 min (1,805 5-s epochs) of crying and 4,372 min (52,464 5-s epochs) of non-crying. The median cry duration of the infants recorded at home was shorter (1.4 min, IQR 0.58–2.6) compared to children recorded during their admission to the hospital (5.8 min, IQR 2.2–16.7). Performance of the algorithm in the independent validation dataset is displayed in Table 1. Overall accuracy was 98.7%, but sensitivity was lower (83.2%) compared to the performance in the training dataset. Due to the relatively low crying incidence compared to non-crying incidence, the specificity of 99.2% led to a PPV of 75.2%. Supplementary Figure 5 displays individual timelines for each infant, displaying the epochs where crying- and misclassifications were present. After post-processing of cry epochs into cry sequences, the median number of cry sequences per infant in the validation dataset was 3 (IQR 1–3), for a total of 39 cry sequences. The median difference between true and predicted cry sequences was 1 (IQR 0.25–1). Furthermore, the median difference between true and predicted cry sequences duration was 6 min (IQR 2–15 min, Table 2). Individual timelines and concordance between true and predicted cry sequences are displayed in Figure 1.

**Table 2:** Individual algorithm performance.

**Characteristics**		**Cry epochs**						**Cry sessions**			
**Subject**	**Duration (min)**	**Annotated count (*n*)**	**Algorithm count (*n*)**	**Sensitivity (%)**	**Specificity (%)**	**PPV (%)**	**NPV (%)**	**Annotated cry sequence count (*n*)**	**Algorithm cry sequence count (*n*)**	**Annotated cry sequence duration**	**Algorithm cry sequence duration**
**Hospital dataset**											
1	764	145	120	80	99.5	66.2	99.7	3	5	37	59
2	610	65	43	90.7	99.6	60	99.9	3	3	19	21
3	245	12	11	90.9	99.9	83.3	99.9	1	1	5	6
4	648	52	20	80	99.5	30.7	99.5	3	3	17	25
5	540	17	12	91.7	99.9	64.7	99.9	1	1	7	8
6	317	721	711	82.3	95.6	81.1	95.9	7	7	117	122
7	16.5	26	24	87.5	97.1	80.7	98.2	1	1	6	8
8	441	200	158	66.5	98.2	52.5	98.9	7	8	55	72
9	77.5	70	80	75	98.8	85.7	97.7	3	3	18.5	26
10	356	99	79	62	98.8	49.5	99.3	3	3	22	36
11	452	320	290	87.9	98.7	79.7	99.3	6	7	64	80
**Home dataset**											
12	36	38	40	95	100	100	99.5	1	1	2.8	2.4
13	13	7	7	100	100	100	100	0	0	0	0
14	2	25	25	100	100	100	100	0	0	0	0
15	1	8	8	100	100	100	100	0	0	0	0

### Algorithm Robustness

**Figure 2:**
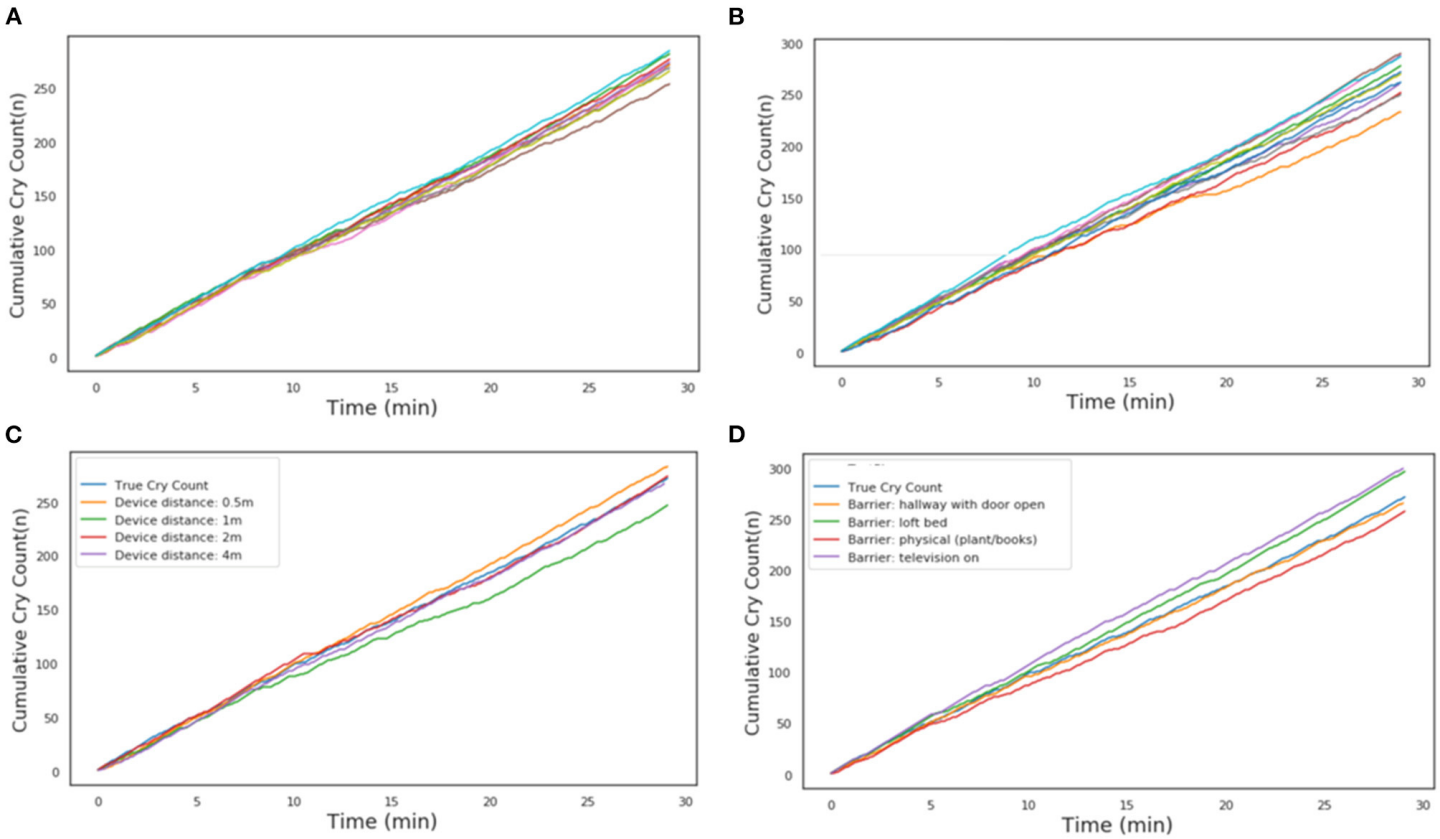
Cumulative cry count during robustness tests. **(A)** Intra-device repeatability. Each individual line is a different run with the same phone. **(B)** Inter-device repeatability. Each individual line is a run with a different phone of the same type. **(C)** Influence of device distance from the audio source. **(D)** Influence of physical barrier or ambient background noise. In each of the panels, the light-blue line is the reference from the audio file.

To ensure the algorithm and smartphone application performs sufficiently for the intended use, multiple tests were conducted to test robustness with the resulting smartphone application. Figure 2A shows the estimated repeatability of the algorithm by repeatedly classifying the same recording with the same device. Figure 2B shows the cumulative cry count of 8 different devices of the same type, which gives an indication of the repeatability. The distance from the audio source, up to 4 meters, did not appear to impact the accuracy of the algorithm (Figure 2C). Finally, blocking the audio signal by placing the phone behind several physical barriers in front of the audio source demonstrated comparable accuracy across conditions (Figure 2D). Creating additional background noise generated by a television appeared to slightly decrease the specificity of the algorithm, as the final cry count according to the algorithm was higher compared to the true number of cries in the audio file.

## Discussion

This paper describes the development and validation of a smartphone-based cry detection algorithm in infants. A random forest classifier had the highest accuracy in the training dataset and achieved a 98.7% accuracy in an independent validation set. Although the sensitivity of 83.2% was slightly lower compared to the estimated accuracy in the training dataset, the individual classification timelines show that this should not lead to unreliable estimation of cry duration. The fact that most misclassifications occurred directly before or after crying indicates that such misclassifications may be due to cry-like fussing, which are difficult to classify for both the algorithm and the human annotators. Post-processing of the detected cry epochs into cry sequences decreased the mismatch and resulted in excellent performance for each individual infant.

The observed accuracy of the algorithm is comparable to others described in the literature, although there is large variation in reported accuracy. Traditional machine learning classifiers and neural network-based classifiers have been used for infant cry analysis and classification (21). We found that several studies that explored the use of minimum, maximum, mean, standard deviation and the variance of MFCCs and other audio features to differentiate normal, hypo-acoustic and asphyxia types using the Chillanto database (6). Support Vector Machines (SVM) are among the most popular infant classification algorithms and routinely outperform neural network classifiers (22, 23). Furthermore, Osmani et al. have illustrated that boosted and bagging trees outperform SVM cry classification (24). Additionally, sensitivities between 35 and 90% with specificities between 96 and 98% have been reported using a convoluted neural network approach (10, 14). Ferreti et al. and Severini et al. also used a neural network approach and achieved a reported precision of 87 and 80%, respectively (11, 12). However, algorithms often lack validation in an independent dataset as, and real-life performance in new and challenging environments will most likely be lower. Our algorithm has several advantages compared to other approaches that have been described in the past. Most importantly, the algorithm was validated on independent and real-life data obtained from two settings where the application could be used in the future. Validation invariably leads to a drop in accuracy compared to the performance of the training data but gives reassurance regarding the generalizability of the algorithm in new settings that were not included during training. Furthermore, the algorithm can be deployed on all Android smartphones and no additional equipment is needed for acquiring the acoustic features. Although it is possible to implement complex deep learning algorithms on portable devices, we demonstrated that a shallow learning algorithm such as a random forest achieves good classifying capability. This means that audio processing and classification can be performed on the device in real-time with the MORE® application, and thus, precludes direct transmission of audio to a central location with inherent preservation of privacy. Finally, the manual feature selection that was performed should lead to further generalizability of the algorithm in new condition, since the observed variability in the excluded audio features would most likely result in a drop in accuracy in challenging acoustic environments. While automated feature selection methods could have been used, automated feature selection requires a static definition of similarity between distributions within features. This is not a straightforward task. Given the nature of the features, we chose to manually exclude features that presented a clearly different distribution from the rest of the features.

All in all, the performance of the algorithm in combination with the mentioned advantages indicate reliability of the algorithm, and may be preferable over manual tracking of cry duration through a diary in several situations. Although the literature regarding sources of inaccuracy in cry monitoring *via* a diary is sparse, several factors make manual tracking through a diary a subjective assessment (5). Observer bias can cause parents to overestimate the true duration of crying, and placebo-effects may cause parents to underestimate true cry duration after an intervention (25). Additionally, parents may underreport nocturnal cry duration when they sleep through short cry sequences during the night. Current tracking of cry duration in clinical settings is performed by nurses, who have other clinical duties as well, possibly making the quality of the cry diary dependent on the number of patients under their care. While the consequences of all of these factors are not easy to quantify, the combination of these sources of inaccuracy leads to the conclusion that objective and automated cry-monitoring could significantly improve the reliability of objective follow-up of cry duration in both clinical trials and -care.Still, parental report of cry duration and cry behavior will remain an important component of follow-up.

A technical limitation of any Android application, including the MORE® application, is that continuous recording can be interrupted by other smartphone applications apps that also access the microphone, like phone calls. However, using a dedicated smartphone for the purpose of cry monitoring will diminish this limitation. Only Motorola G5/G6 phones were used during each phase of algorithm development and validation. Although performance on other smartphones is uncertain, the approach used in this paper could easily be replicated to adapt the algorithm to other devices and obtain a similar accuracy. In the future, incorporation of covariates such as age, sex or location in the model may improve classifying capability even further, and further stratification could allow to discriminate different types of crying. In this manner cries from asphyxiated infants (26), pre-term infants (27), or infants with respiratory distress syndrome could be differentiated from healthy infants (13). One potential technical limitation of our approach is the use of loudspeakers to create the training dataset. An ideal training dataset would include smartphone-based audio recordings of multiple subjects under different conditions over a long period of time. We found the most appropriate alternative was to re-record open-sourced cry corpus using smartphone. While the playback could have potentially hindered the quality of the openSmile features and thus the classification, it resulted in excellent classification performance of the home and hospital recordings. Hence the impact of the quality of the loudspeaker-based dataset was deemed acceptable. A follow-up study that uses an original smartphone-based cry corpus could potentially improve the accuracy of the classification algorithm. The start- and stop criteria used to determine the beginning and end of a cry sequence are a new proposal that was not previously described in the literature. However, the criteria appear reasonable and individual timeline figures demonstrated that this post-processing step was able to generate a solid high-level overview of individual cry behavior. Still, alternative criteria could obtain similar accuracy and may be explored in the future.

The developed algorithm already provides an excellent overview of the cry behavior of infants and preliminary tests of the robustness of the resulting algorithm show inter- and intradevice repeatability and reliability up to 4 m from the audio source. The algorithm can replace current methods to track cry behavior, such as cry diaries, in clinical and at-home settings. However, more research is needed before implementing the cry duration and the amount of cry sequences as digital endpoint in trials. Clinical validation of cry duration and cry sequence count as digital biomarker in a patient population is necessary, and should focus on establishing new normative values for objectively determined cry- and sequence duration and -count, the difference between patients and healthy controls, correlation with disease-severity and sensitivity to change after an intervention (28).

## Conclusion

The proposed smartphone-based algorithm is accurate, robust to various conditions and has the potential to improve clinical follow-up of cry behavior and clinical trials investigating interventions to enhance infant well-being.

## Data Availability Statement

The raw data supporting the conclusions of this article will be made available by the authors, without undue reservation.

## Ethics Statement

The studies involving human participants were reviewed and approved by METC LDD Post-zone P5-P, Kamer P5-22 Post-bus 9600 2300 RC Leiden 071 526 3241/071 526 6963 metc-ldd@lumc.nl. Written informed consent to participate in this study was provided by the participants' legal guardian/next of kin.

## Author Contributions

AZ performed the analysis and wrote the manuscript. MK conceptualized the study, supported data collection, and wrote the manuscript. MG and ED collected data, supported the analysis, and co-wrote the manuscript. VE, R-JD, and FS supervised and provided input for the analysis and reviewed the manuscript. GD conceptualized the study, supervised the clinical part of the study, and reviewed the manuscript. AC conceptualized the study, provided input for the analysis, and reviewed the manuscript. All authors contributed to the article and approved the submitted version.

## Conflict of Interest

The authors declare that the research was conducted in the absence of any commercial or financial relationships that could be construed as a potential conflict of interest.
