# Supplementary material for: Development and Technical Validation of a Smartphone-Based Cry Detection Algorithm
Source: Front Pediatr. 2021 Apr 13;9:651356. doi: 10.3389/fped.2021.651356 (PMC8076575; doi:10.3389/fped.2021.651356)

Supplementary Figure S1. Schematic overview of analysis steps

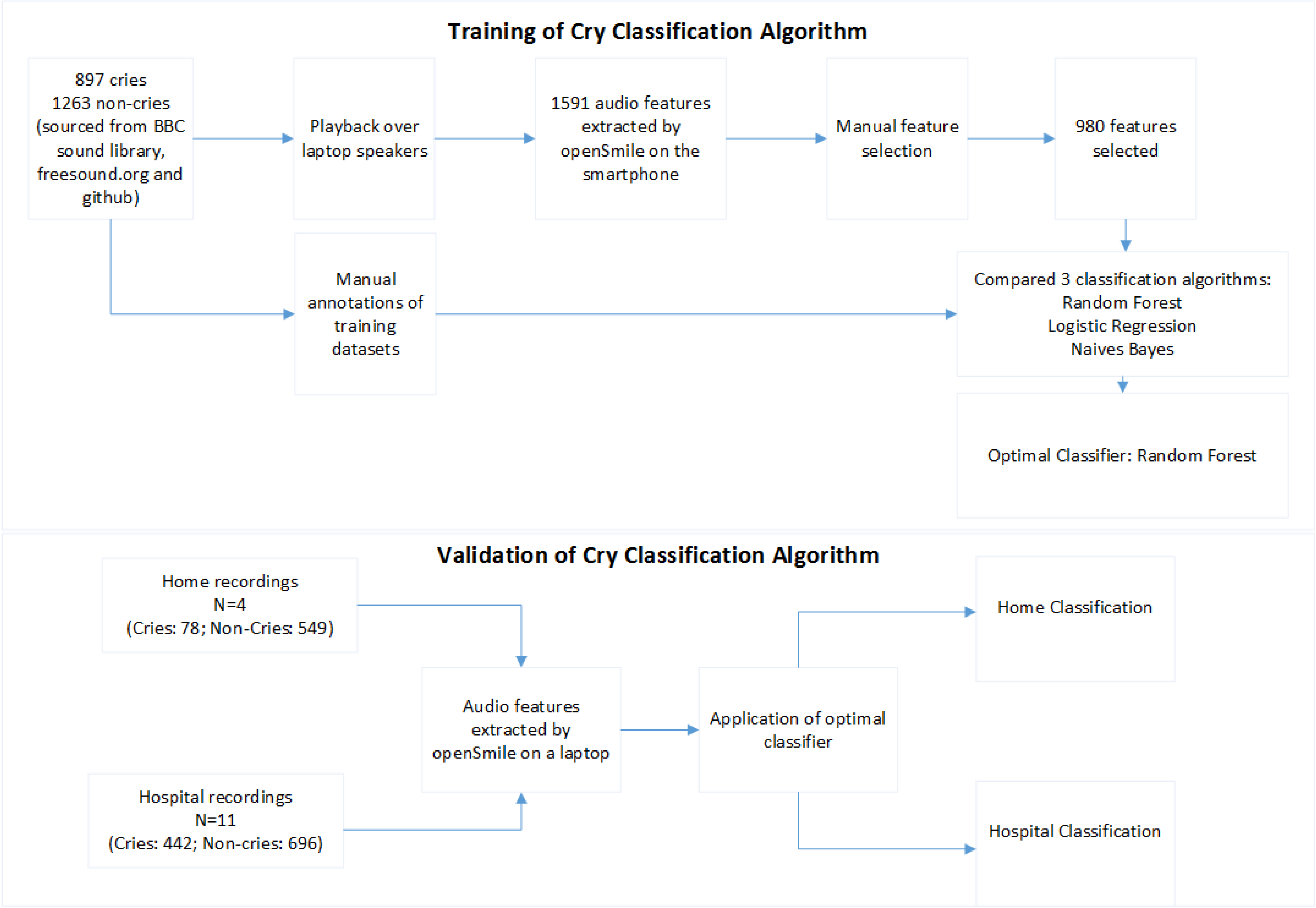

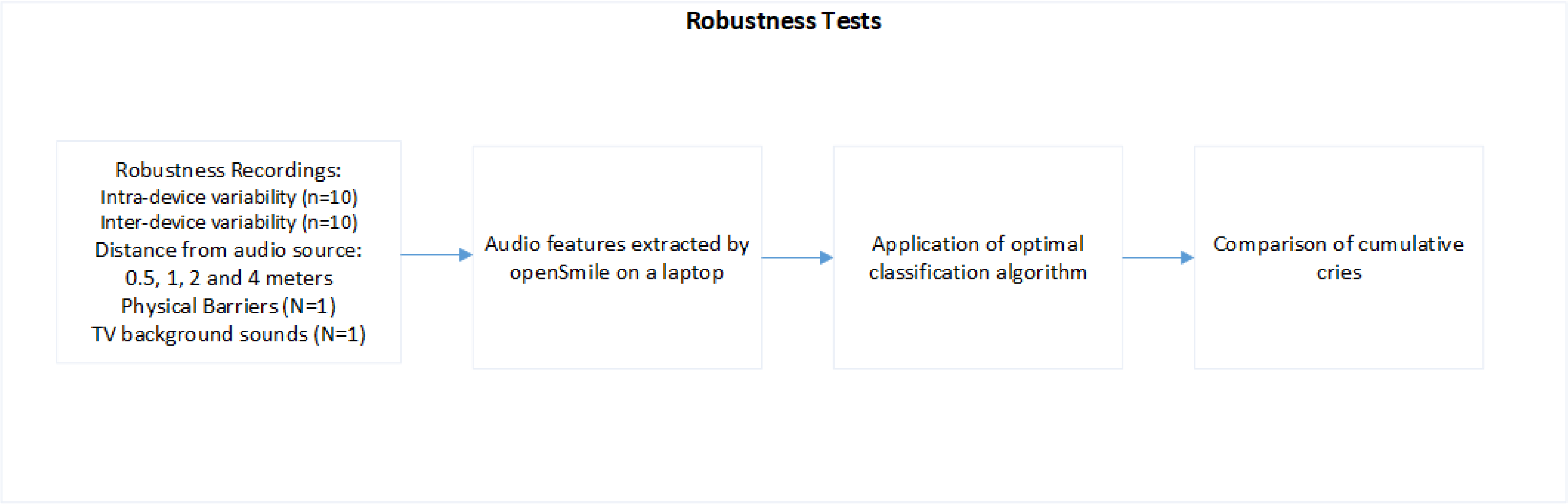
Robustness Tests
Robustness Recordings:
Intra-device variability (n=10)
Inter-device variability (n=10)
Distance from audio source:
0.5, 1, 2 and 4 meters
Physical Barriers (N=1)
TV background sounds (N=1)
Audio features extracted by openSmile on a laptop
Application of optimal classification algorithm
Comparison of cumulative cries

Supplementary Table S2. Audio sources

Dataset	Source	Crying (5-second epochs)	Non-crying** (5-second epochs)
Training Dataset	Github repository:*** https://github.com/giulbia/baby_cry_detection	146	216
	Freesound.org: *** https://freesound.org/search/?q=infant+cry	102	15
	British Broadcasting Company sound library: *** https://sound-effects.bbcrewind.co.uk/	207	336
Home Validation Dataset	Home Recordings	78	549
Hospital Validation Dataset	Hospital recordings	350	594
	Merged epochs*	92	102
	Total	975	1812

*** Merged crying sounds with additional background noise**

**** The non-crying sound included common baby sounds (babies hiccoughing, gurling, babbling and yelleing), common human sounds (breathing, coughing, talking), general indoor sounds (doors closing, footsteps and vacuuming) and general outdoor sounds (birds, thunder, sirens).**

***** This is a labelled collection of environmental audio recordings. The audio recordings have been extracted from public field recordings.**

Supplementary Text S3. Audio features and feature selection

OpenSMILE generated features from each 5 second epoch in the following domains:

Feature group	Description
Fundamental frequency (F0)	Pitch
Jitter and shimmer	Voice quality
Mel-frequency cepstrum (coefficients)	Power spectrum
Line spectral frequencies	Frequencies
Loudness	Sum of auditory spectrum. (Intensity & approximate loudness)
Voicing	Probability of voicing

For each domain, the following statistics were derived by the openSMILE software:

Statistics obtained from each feature during each 5-second epoch
Arithmetic mean
Quartiles and IQR ranges (1-2, 1-3, 2-3)
Skewness and kurtosis
Linear regression slope, offset and approximation error
Relative position of minimum and maximum
Percentile 1%, percentile 99% and range
Standard deviation
Percentage of frames above 75/90% of range

Feature selection

Supplementary Figure S3a

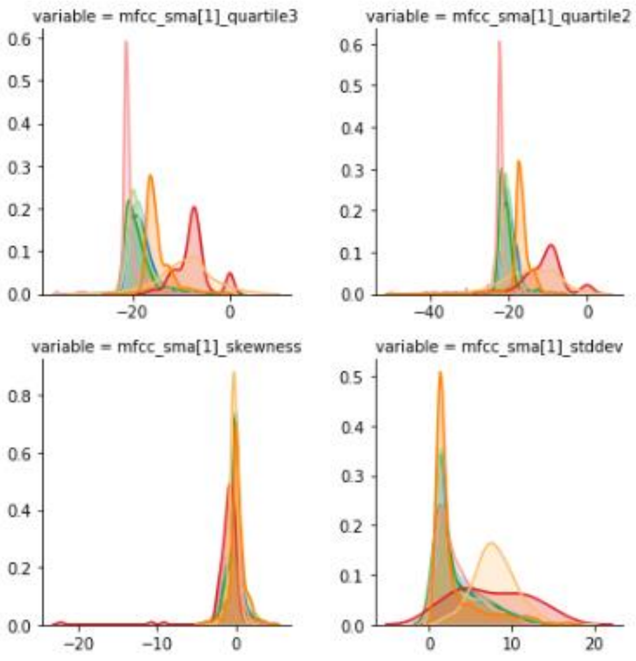

Example of distribution plots of each feature used during the feature selection process. Each color represents a different condition. Of the displayed features, only the bottom left feature (mfcc_sma[1] skewness) was included in the final dataset.

Supplementary Figure S4. Variable importance

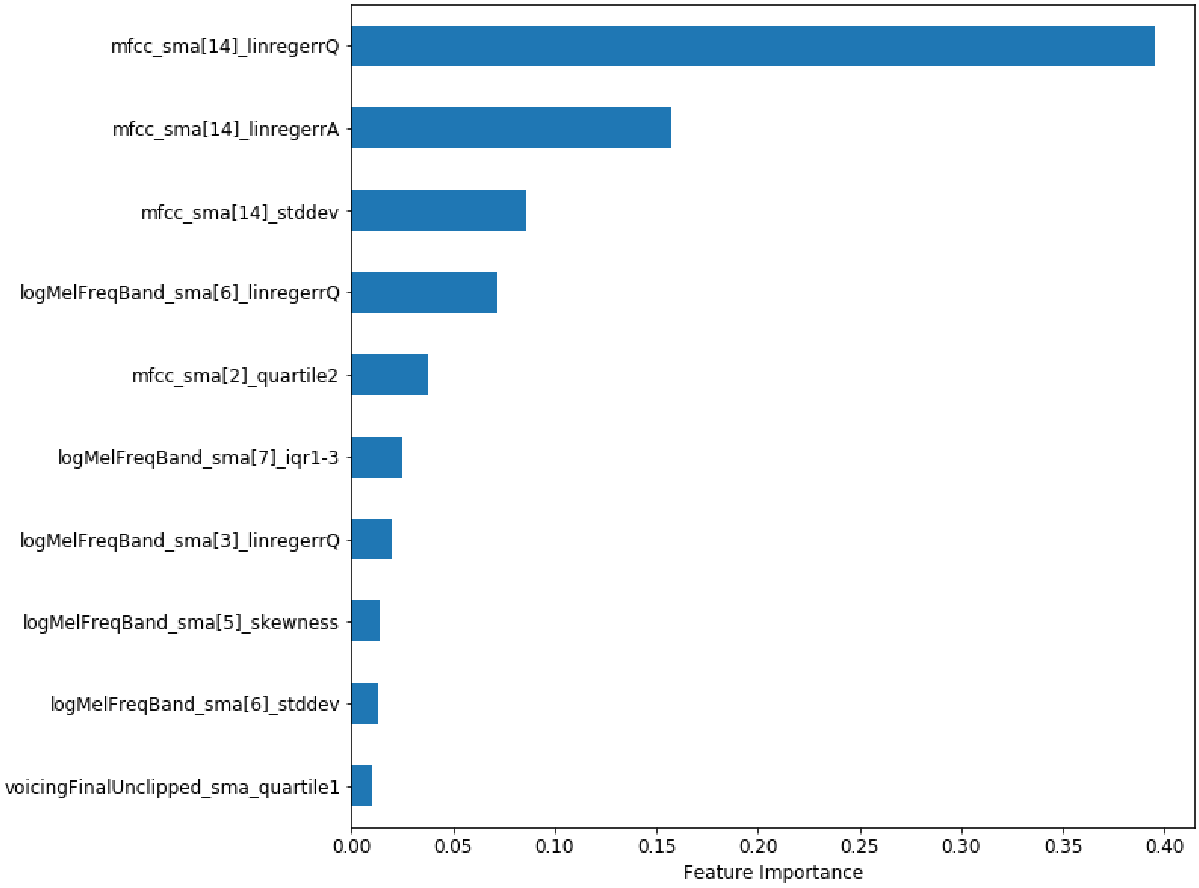

Feature importance plot of the final algorithm. On the y-axis, the 10 most important features derived from the openSMILE software are displayed. The bars and the x-axis represent the relative importance of each feature.

Supplementary Figure S5. True and predicted crying epochs per infant

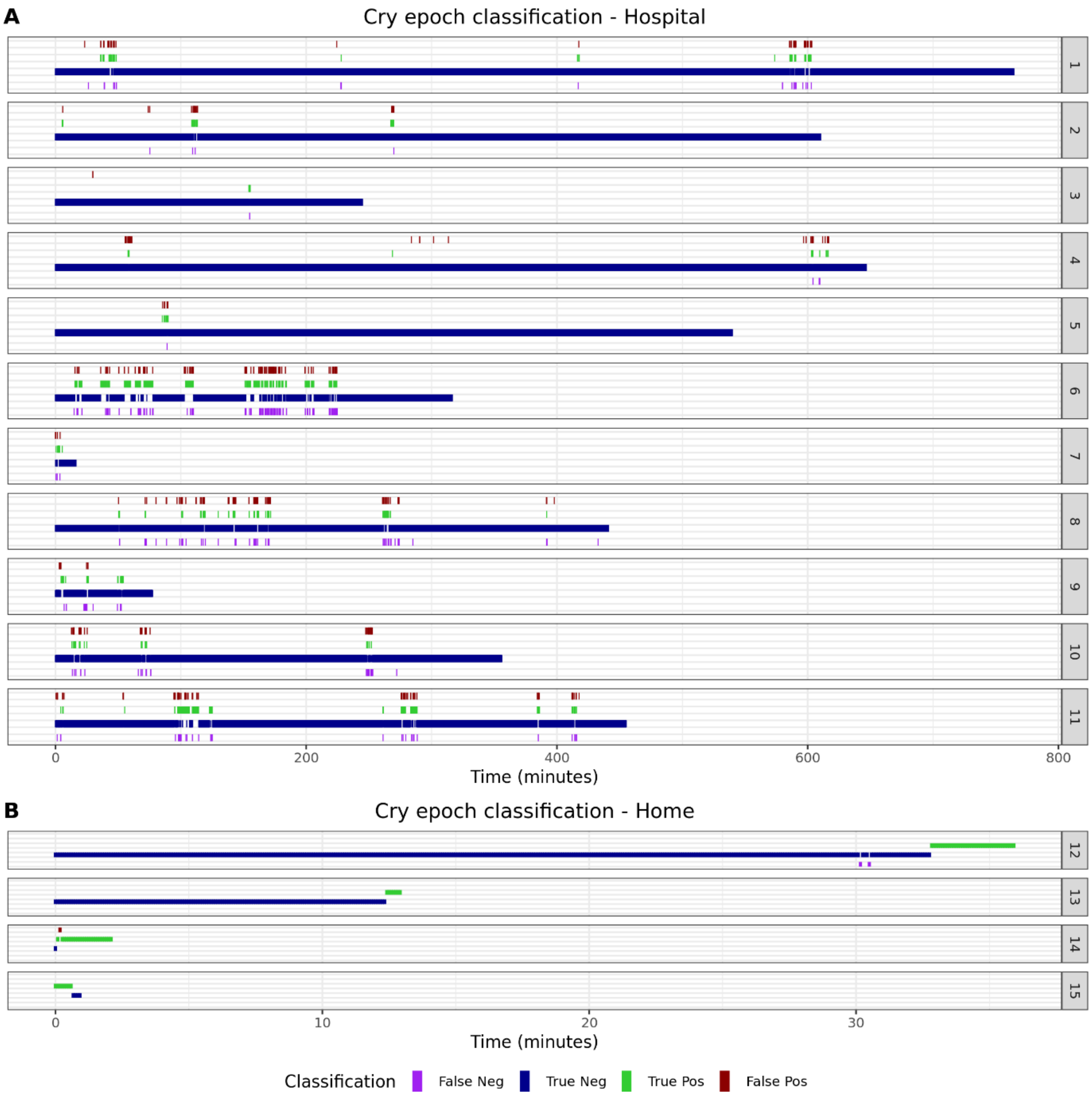

Supplement: Supplementary file 1 [file Data_Sheet_1.PDF]
